# Comparison of 13-, 15- and 20-valent pneumococcal conjugate vaccines in the paediatric Canadian population: A cost-utility analysis

**DOI:** 10.14745/ccdr.v51i23a02

**Published:** 2025-02-12

**Authors:** Alison E Simmons, Gebremedhin B Gebretekle, Robert Pless, Aleksandra Wierzbowski, Matthew Tunis, Ashleigh R Tuite

**Affiliations:** 1 Infectious Diseases and Vaccination Program Branch, Public Health Agency of Canada, Ottawa, ON; 2 Dalla Lana School of Public Health, University of Toronto, Toronto, ON

**Keywords:** pneumococcal disease, vaccination, cost-utility analysis, health economics, modelling

## Abstract

**Background:**

Two pneumococcal conjugate vaccines, covering 15 and 20 *Streptococcus pneumoniae* serotypes (Pneu-C-15 and Pneu-C-20, respectively), were recently approved for use in the Canadian paediatric population.

**Objective:**

To assess the cost-effectiveness of Pneu-C-15 and Pneu-C-20 in unvaccinated infants initiating routine pneumococcal vaccination, compared to the currently used 13-valent conjugate vaccine (Pneu-C-13).

**Methods:**

A static cohort model was used to estimate sequential incremental cost-effectiveness ratios (ICERs in 2022 Canadian dollars per quality-adjusted life year [QALY]) of Pneu-C-13, Pneu-C-15 and Pneu-C-20 in the paediatric population starting their primary series. Costs and outcomes were calculated over a 10-year time horizon at the program level and a lifetime time horizon at the individual level and discounted at a rate of 1.5% per year. We explored the impact of uncertainties in model parameters and assumptions in scenario and sensitivity analyses.

**Results:**

Routine use of Pneu-C-20 and, to a lesser extent, Pneu-C-15 is projected to reduce pneumococcal disease burden, compared to Pneu-C-13. Based on product cost assumptions, sequential ICERs for Pneu-C-15 and Pneu-C-20 were $58,800 and $135,200 per QALY gained from the health system perspective and $18,272 and $93,416 per QALY gained from the societal perspective, excluding indirect effects. A reduction in serotype-attributable disease due to indirect vaccine effects of 5% or greater resulted in ICERs below $30,000 per QALY gained for Pneu-C-15 and Pneu-C-20, with the optimal strategy determined by the magnitude and time to reach a reduction in pneumococcal disease.

**Conclusion:**

Both Pneu-C-15 and Pneu-C-20 are expected to increase QALYs in Canadian children compared to Pneu-C-13 and may be cost-effective interventions.

## Introduction

Pneumococcal disease (PD), caused by *Streptococcus pneumoniae*, causes significant global morbidity and mortality, particularly in children, older adults and people with immunocompromising conditions. Although *S. pneumoniae* frequently colonizes the human nasopharynx without causing illness, it can cause severe invasive (e.g., meningitis and bacteremia) and, more commonly, non-invasive (e.g., pneumococcal community acquired pneumonia [pCAP] and acute otitis media [AOM]) disease ((1)). More than 100 distinct capsular types, or serotypes, of *S. pneumoniae* have been identified, but the majority of invasive pneumococcal disease (IPD) cases are attributed to a subset of these serotypes ((2,3)).

Infectious disease modelling is often used to support pneumococcal vaccine decisions due to complex serotype dynamics observed over years under previous vaccination schedules. In the early 2000s, the first pneumococcal conjugate vaccines (Pneu-C-7 and Pneu-C-10) were authorized for use in Canada and were provided in publicly funded immunization programs. In 2009, Pneu-C-13 vaccine received approval and in 2010, Canada’s National Advisory Committee on Immunization (NACI) recommended that healthy children receive 2+1 doses of Pneu-C-13 at two, four and 12–15 months of age or 3+1 doses of Pneu-C-13 at two, four, six and 12–18 months of age ((4)). The Pneu-C-13 vaccine consists of serotypes 1, 3, 4, 5, 6A, 6B, 7F, 9V, 14, 18C, 19A, 19F and 23F. Two pneumococcal conjugate vaccines, covering 15 and 20 *S. pneumoniae* serotypes, were authorized by Health Canada for use in paediatric populations on July 8, 2022 (Pneu-C-15) and July 21, 2023 (Pneu-C-20). The Pneu-C-15 vaccine includes Pneu-C-13 serotypes as well as serotypes 22F and 33F, and Pneu-C-20 includes Pneu-C-13 serotypes as well as serotypes 8, 10A, 11A, 12F, 15B, 22F and 33F ((5,6)).

Following the introduction of Pneu-C-13, the incidence of IPD caused by the 13 *S. pneumoniae* serotypes included in the vaccine decreased across all age groups ((7–9)); however, overall IPD incidence remained relatively unchanged across all age groups due to *S. pneumoniae* serotype replacement as well as persistence of some Pneu-C-13 serotypes ((10,11)). Between 2016 and 2020, a significant increase in IPD caused by serotypes 19F and 11A was observed among children younger than five years old in Canada ((12)). Serotype 19F is included in Pneu-C-13, Pneu-C-15 and Pneu-C-20, and serotype 11A is included only in Pneu-C-20.

Given the broader serotype coverage provided by Pneu-C-15 and Pneu-C-20, we conducted a model-based economic evaluation to assess the cost-effectiveness of their use in the Canadian paediatric population compared to the current standard of care.

## Methods

We developed a static Markov cohort model to quantify the health impact of three paediatric pneumococcal vaccination strategies in previously unvaccinated infants. We compared 2+1 doses of Pneu-C-13 (current policy), Pneu-C-15 and Pneu-C-20. *Streptococcus pneumoniae-*associated health outcomes from the cohort model were used to inform a cost-utility analysis. Outcomes included the incidence of IPD, non-invasive pCAP and AOM, hospitalizations, deaths, costs, quality-adjusted life years (QALYs) and incremental cost-effectiveness ratios (ICERs). At the time of the analysis, NACI had not yet published recommendations for the use of Pneu-C-15 and Pneu-C-20 in the paediatric population. This economic analysis was conducted to support the development of NACI’s recommendations and additional details of the economic evidence considered are available online ((13)).

### Model structure

**Figure 1:**
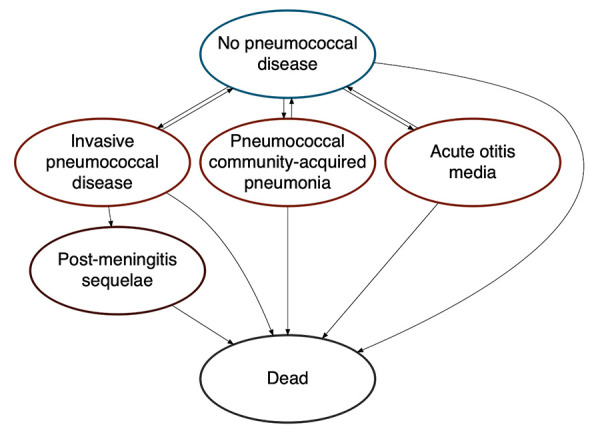
Pneumococcal disease model health states^a,b^ ^a^ Vaccinated and unvaccinated people experienced the same health states, with risk modified based on vaccination status, type of vaccine received and time since vaccination ^b^ Post-meningitis sequelae include auditory and neurologic sequelae

Our model followed a multi-age, open population cohort over 10 years. Birth and death rates within the cohort were informed by Canadian population projections ((14–16)). Individuals were free of PD at model entry, but could develop IPD, pCAP and AOM over their lifetime (**Figure 1**, **Table 1**). A subset of individuals with IPD developed post-meningitis sequelae. We assumed IPD was treated in an inpatient setting, pCAP was treated in an inpatient or outpatient setting and AOM was treated in an outpatient setting. Incidence, costs and health consequences of AOM were restricted to individuals younger than 10 years of age ((17)).

**Table 1:** Epidemiologic parameters

Parameter	Base	Range	Reference(s)
**IPD incidence (per 100,000 population)**			
Younger than 2 years	14.5	-	CNDSS, 2019; ICS program, 2019 ((18))
2–4 years	10.2	-	
5–17 years	2.1	-	
18–49 years	5.2	-	
50–64 years	13.6	-	
65 years and older	23.8	-	
**CAP incidence (per 100,000 population)**			
Younger than 5 years	4,991.1	-	Nasreen *et al.*, 2022 ((19))
5–17 years	1,249.0	-	
18–39 years	815.9	-	
40–64 years	1,529.9	-	
65–74 years	3,095.7	-	
75–84 years	5,398.1	-	
85 years and older	10,122.7	-	
**AOM incidence (per 100,000 population)**			
Younger than 5 years	25,467.6	-	Nasreen *et al.*, 2022 ((19))
5–17 years	7,225.9	-	
18–39 years	2,204.4	-	
40–64 years	2,058.6	-	
65–74 years	1,954.7	-	
75–84 years	1,857.4	-	
85 years and older	1,621.4	-	
**Relative risk of PD in higher incidence setting**			
Younger than 2 years	6.8	-	CNDSS, 2019; ICS program, 2015–2019 ((18))
2–4 years	0.9	-	
5–17 years	3.9	-	
18–49 years	2.1	-	
50–64 years	2.1	-	
65 years and older	2.4	-	
**Proportion of patients with CAP attributed to *S. pneumoniae* (%)**			
Younger than 1 year	6.0	5.1–9.1	King, 2023; LeBlanc *et al.*, 2022; Pneumonia Etiology Research for Child Health (PERCH) Study Group, 2019 ((20–22))
1–15 years	12.0	10.1–18.2	
16–49 years	19.5	17.3–21.7	
50–64 years	19.0	17.3–20.7	
65 years and older	11.2	10.4–12.1	
**Proportion of patients with AOM attributed to *S. pneumoniae* (%)**			
Younger than 18 years	17	14–22	Kim *et al.*, 2017; King, 2023 ((20,23))
**Proportion of patients with pCAP managed in inpatient setting (%)**			
Younger than 65 years	4.6	2.2–9.3	O’Reilly *et al.*, 2023 ((24))
65 years and older	12.3	7.9–18.6	
**Proportion of patients with IPD with meningitis (%)**			
Younger than 1 year	16.9	13.3–21.1	Morrow *et al.*, 2007 ((17))
1–4 years	4.6	3.0–6.8	
5–9 years	8.7	4.1–15.9	
10–19 years	8.5	5.1–13.3	
20–64 years	5.1	3.9–6.4	
65 years and older	3.1	2.2–4.1	
**Proportion of patients with meningitis with long-term post-meningitis sequelae (%)**			
Neurologic sequelae	12.2	5.3–19.1	Jit, 2010 ((25))
Hearing loss	8.2	4.5–11.9	
**Proportion of patients with AOM with ear tube placement (%)**			
Younger than 10 years	6	4–12	Canadian Institute for Health Information, 2020; Chuck *et al.*, 2010; Nasreen *et al.*, 2022; Assumption ((19,26,27))
**IPD case fatality (%)**			
Younger than 1 year	11.8	11.2–12.3	Wijayasri *et al.*, 2019 ((7))
1–4 years	1.6	0.8–2.7	
5–49 years	5.7	4.9–6.7	
50–64 years	10.9	9.9–12	
65 years and older	17.2	16.2–18.3	
**pCAP (inpatient) case fatality (%)**			
Younger than 10 years	1.0	0.3–3.1	LeBlanc *et al.*, 2022; Morrow *et al.*, 2007; Assumption ((17,22))
10–15 years	1.6	0.6–4.3	
16–49 years	3.8	1.7–7.0	
50–64 years	4.8	2.7–7.1	
65 years and older	9.9	7.7–12.3	
**Vaccine-type serotype distribution (%), younger than 2 years**			
ST3	8	-	National Microbiology Laboratory, 2019 ((18))
Pneu-C-13/non-ST3	9	-	
Pneu-C-15/non-Pneu-C-13	21	-	
Pneu-C-20/non-Pneu-C-15	19	-	
NVT	43	-	
**Vaccine-type serotype distribution (%), 2–4 years**			
ST3	11	-	National Microbiology Laboratory, 2019 ((18))
Pneu-C-13/non-ST3	16	-	
Pneu-C-15/non-Pneu-C-13	16	-	
Pneu-C-20/non-Pneu-C-15	23	-	
NVT	33	-	
**Vaccine-type serotype distribution (%), 5–17 years**			
ST3	8	-	National Microbiology Laboratory, 2019 ((18))
Pneu-C-13/non-ST3	23	-	
Pneu-C-15/non-Pneu-C-13	20	-	
Pneu-C-20/non-Pneu-C-15	14	-	
NVT	35	-	
**Vaccine-type serotype distribution (%), 18–49 years**			
ST3	10	-	National Microbiology Laboratory, 2019 ((18))
Pneu-C-13/non-ST3	32	-	
Pneu-C-15/non-Pneu-C-13	11	-	
Pneu-C-20/non-Pneu-C-15	21	-	
NVT	26	-	
**Vaccine-type serotype distribution (%), 50–64 years**			
ST3	12	-	National Microbiology Laboratory, 2019 ((18))
Pneu-C-13/non-ST3	32	-	
Pneu-C-15/non-Pneu-C-13	11	-	
Pneu-C-20/non-Pneu-C-15	21	-	
NVT	26	-	
**Vaccine-type serotype distribution (%), 65 years and older**			
ST3	13	-	National Microbiology Laboratory, 2019 ((18))
Pneu-C-13/non-ST3	16	-	
Pneu-C-15/non-Pneu-C-13	15	-	
Pneu-C-20/non-Pneu-C-15	14	-	
NVT	42	-	

Abbreviations: AOM, acute otitis media; CAP, community acquired pneumonia; CNDSS, Canadian Notifiable Disease System; ICS, International Circumpolar Surveillance; IPD, invasive pneumococcal disease; NVT, non-vaccine type; pCAP, pneumococcal community acquired pneumonia; PD, pneumococcal disease; Pneu-C, pneumococcal conjugate vaccine; ST3, serotype 3; -, not applicable

Upon model entry, a proportion of each birth cohort was vaccinated at two, four and 12 months of age, based on estimated Pneu-C-13 vaccination coverage (**Table 2**) ((28)). Vaccination was assumed to reduce the risk of PD caused by the serotypes included in the vaccine. We assumed vaccine effectiveness (VE) for Pneu-C-15 and Pneu-C-20 was equivalent to VE for Pneu-C-13. All vaccines had a lower VE against serotype 3 compared to the other vaccine serotypes. In the model, vaccine-derived protection began after the second dose and waned over 15 years ((29)). The base case model did not include indirect effects of vaccination including herd immunity and serotype replacement.

**Table 2:** Vaccination parameters

Parameter	Base	Range	Reference(s)
**Vaccination coverage (%)**			
2 doses	87	-	Assumption^a^
2+1 doses	84.5	-	Childhood National Immunization Coverage Survey (cNICS), 2022 ((28))
**Pneu-C effectiveness of 2+1 doses (%)**			
VT-IPD	85	67–96	Farrar *et al.*, 2022; Prasad *et al.*, 2023; Assumption ((29,30))
ST3-IPD	33	10–66	Farrar *et al.*, 2022; Prasad *et al.*, 2023; Assumption ((29,30))
VT-pCAP	64	50–72	Prasad *et al.*, 2023; Stoecker, 2023; Assumption (based on adult data for relative VE for IPD vs. pCAP) ((29,31))
ST3-pCAP	25	19–28	Assumption (based on IPD)
VT-AOM	54	40–64	Eskola, 2001 ((32))
ST3-AOM	21	15–25	Assumption (based on IPD)
**Pneu-C effectiveness of 2 doses**			
% of VE achieved with first 2 doses of series	75	60–90	Andrews *et al.*, 2014; Assumption ((33))
**Duration of protection**			
Pneu-C	15 years: stable for 5 years, linear decline to 0 over 10 years	-	Prasad *et al.*, 2023 ((29))

Abbreviations: IPD, invasive pneumococcal disease; pCAP, pneumococcal community acquired pneumonia; Pneu-C, pneumococcal conjugate vaccine; ST3-AOM, serotype 3 acute otitis media; ST3-IPD, serotype 3 invasive pneumococcal disease; ST3-pCAP, serotype 3 pneumococcal community acquired pneumonia; VE, vaccine effectiveness; VT-AOM, vaccine-type acute otitis media; VT-IPD, vaccine-type invasive pneumococcal disease; VT-pCAP, vaccine-type pneumococcal community acquired pneumonia; -, not applicable

^a^ Based on diphtheria, tetanus and acellular pertussis (DTaP) coverage at 3 months (1 or more doses) and 12 months (3 or more doses) ((28))

### Cost-utility analysis

We used the outputs from our model to inform a cost-utility analysis of the three vaccination strategies over a 10-year programmatic time horizon. A lifetime time horizon was used at the individual level (i.e., all long-term consequences of PD accrued over an individual’s lifetime were included). The assumed cost per dose in our base case was $71.50 for Pneu-C-13, $78.10 for Pneu-C-15 and $90.10 for Pneu-C-20 (**Table 3**). An unpublished analysis conducted by the Public Health Agency of Canada found that Canadian negotiated vaccine prices across all vaccine programs are typically 30%–50% of United States contract prices; we applied a 40% discount rate to the United States’ Centers for Disease Control and Prevention public vaccine prices to estimate the cost per dose in our base case ((34)). Costs and utilities were derived preferentially from Canadian surveillance data and published studies, and by assumption (Table 3, **Table 4**). We applied a discount rate of 1.5% to QALYs and costs, with costs inflated to 2022 Canadian dollars ((35)). Probabilistic model estimates were based on 10,000 simulations. For each model simulation, parameters were drawn from distributions and results were calculated for each scenario; summary results across the 10,000 simulations were computed. Values with ranges provided in Tables 1-4 indicate model parameters that were sampled probabilistically to capture uncertainty (i.e., sampled from beta distributions for probabilities and utilities and gamma distributions for costs). The model was constructed in R and parameters specifying distributions (shape and scale for gamma distributions and shape1 and shape2 for beta distributions) were estimated using the specified means and ranges ((36,37)). We conducted our analyses from both the health system and societal perspectives. In addition to including health outcome and health system costs, the latter also incorporates costs not paid by the publicly funded health system (e.g., direct out-of-pocket costs, productivity loss) ((38)).

**Table 3:** Direct and indirect cost parameters

Parameter	Base ($)	Range ($)	Reference(s)
**Cost per dose of vaccine**			
Vaccine administration	16.77	12.58–20.96	O’Reilly *et al.*, 2017 ((39))
Pneu-C-13	71.5	-	Centers for Disease Control and Prevention; Assumption ((34))
Pneu-C-15	78.1 (9.2% higher than Pneu-C-13)	72.2–87.9 (1%–23% higher than Pneu-C-13)	
Pneu-C-20	90.1 (26.1% higher than Pneu-C-13)	78.6–107.2 (10%–50% higher than Pneu-C-13)	
**Cost per patient with IPD**			
Younger than 5 years	20,468	17,422–23,755	Discharge Abstract Database, 2015–2019 ((40–43))
5–17 years	14,717	12,510–17,100	
18–49 years	28,812	26,559–31,155	
50–64 years	29,146	27,363–30,984	
65–74 years	28,955	26,727–31,271	
75 years and older	21,501	20,001–23,054	
**Cost per patient with pCAP managed in inpatient setting**			
Younger than 18 years	7,345	7,189–7,545	O’Reilly *et al.*, 2023 ((24))
18–64 years	14,185	13,708–14,686	
65 years and older	14,179	13,931–14,433	
**Cost per patient with pCAP managed in outpatient setting**			
Younger than 18 years	450	438–461	O’Reilly *et al.*, 2023 ((24))
18–64 years	1,187	1,154–1,221	
65 years and older	3,343	3,283–3,400	
**Cost per AOM case, excluding ear tube placement**			
Younger than 2 years	260	258–301	Gaboury *et al.*, 2010; Assumption ((44))
2–9 years	178	148–207	
Cost of surgery for ear tube placement	1,790	1,340–2,240^a^	Canadian Institute for Health Information, 2020 ((26))
**Cost of care for patients with post-meningitis sequelae (per year)**			
Annual cost of care for those with auditory sequelae	2,783.3	2,087.5–3,479.2^a^	Christensen *et al.*, 2014 ((45))
Annual cost of care for those with neurologic sequelae	9,262.4	6,946.8–11,578.0^a^	
**Out-of-pocket costs**			
Medication, younger than 65 years	18.1	13.6–22.6	American Academy of Pediatrics, 2021; Metlay *et al.*, 2019; Ontario Ministry of Health, 2022; Patented Medicine Prices Review Board Canada, 2019–2020 ((46–49))
Transportation to inpatient care	139	29–333	Canada Revenue Agency, 2022; Colbert, 2020; Discharge Abstract Database, 2015–2019 ((40–43,50,51))
Transportation to outpatient care	3.7	2.8–4.6^a^	Canada Revenue Agency, 2022; Pong and Pitblado, 2005 ((51,52))
**Relative increase of direct costs in higher cost setting**			
Inpatient case	1.8	-	NACI ((53))
Outpatient case	1.2	-	
Travel for outpatient case	33	-	
**Workdays lost (16 years and older)**			
Inpatient IPD or pCAP	15	9–29	Pasquale *et al.*, 2019 ((54))
Outpatient pCAP	5.4	1.8–6.3	
**Reduction in employment in patients with post-meningitis sequelae (%)**			
Auditory sequelae	25	15–35	Bizier *et al.*, 2016; Jiang *et al.*, 2012 ((55,56))
Neurologic sequelae	98	75–100	Jiang *et al.*, 2012; Assumption ((56))
**Caregiver workdays lost, IPD**			
Younger than 5 years	11.2	9.4–13.0	Discharge Abstract Database, 2015–2019 ((40–43))
5–15 years	9.9	7.8–12.0	
16 years and older	5.4	1.5–10.8	Wyrwich *et al.*, 2015 ((57))
**Caregiver workdays lost, inpatient pCAP**			
Younger than 5 years	4.2	4.2–4.3	Discharge Abstract Database, 2015–2019 ((40–43))
5–15 years	5.0	7.8–12.0	
16 years and older	5.4	1.5–10.8	Wyrwich *et al.*, 2015 ((57))
**Caregiver work days lost, outpatient pCAP**			
Younger than 16 years	5.4	1.8–6.3	Pasquale *et al.*, 2019; Assumption ((54))
16 years and older	1.1	1.0–1.2	Dubé *et al.*, 2011 ((58))
**Caregiver work days lost, AOM**			
AOM	1.3	0.8–1.7	Barber *et al.*, 2014; Dubé *et al.*, 2011 ((58,59))
Ear tube placement	2.1	-	Petit *et al.*, 2003 ((60))
**Caregiver work days lost, sequelae**			
Auditory sequelae (annual)	0	-	Assumption
Neurologic sequelae (annual)	190	146–240^a^	Ganapathy *et al.*, 2015 ((61))
**Caregiver work days lost, vaccination**			
Visit healthcare provider for vaccination	0.5	-	Assumption
**Average employment income ($)**			
Age 16 years and older	Age-specific values	-	Statistics Canada ((62))
Caregiver	58,811	-	
**Labour force participation (%)**			
Age 16 years and older	Age-specific values	-	Statistics Canada ((63))
Caregiver (age 25–54 years)	87	-	

Abbreviations: AOM, acute otitis media; IPD, invasive pneumococcal disease; NACI, National Advisory Committee on Immunization; pCAP, pneumococcal community acquired pneumonia; Pneu-C, pneumococcal conjugate vaccine; -, not applicable

^a^ Range defined as ±25% of the base value

**Table 4:** Health utilities and utility decrements

Parameter	Base	Range	Reference(s)
**Background health utility**			
Younger than 6 years	0.97	0.96–0.98	Molina *et al.*, 2023; Assumption ((64))
6–11 years	0.95	0.94–0.96	Molina *et al.*, 2023 ((64))
12–17 years	0.89	0.87–0.91	Yan *et al.*, 2023 ((65))
18–24 years	0.879	0.863–0.895	
25–34 years	0.881	0.864–0.898	
35–44 years	0.878	0.863–0.893	
45–54 years	0.855	0.838–0.872	
55–64 years	0.839	0.822–0.856	
65–74 years	0.867	0.849–0.885	
75 years and older	0.861	0.835–0.887	
**IPD utility decrement**			
Younger than 19 years	0.028	0.0165–0.0308	Tang *et al.*, 2022; Assumption ((66))
19–64 years	0.0533	0.0425–0.0547	
65 years and older	0.0745	0.0001–0.0745	
**Outpatient pCAP utility decrement**			
Younger than 19 years	0.0004	0.0001–0.0329	Tang *et al.*, 2022 ((66))
19–64 years	0.0094	0.0001–0.0205	
65 years and older	0.0586	0.0271–0.0659	
**Inpatient pCAP utility decrement**			
Younger than 19 years	0.0105	0.001–0.0155	Tang *et al.*, 2022; Assumption ((66))
19–64 years	0.0396	0.0001–0.168	
65 years and older	0.1154	0.0068–0.29	
**AOM utility decrement**			
Younger than 10 years	0.0016	0–0.1461	Tang *et al*., 2022 ((66))
**Auditory sequelae utility decrement (per year)**			
Younger than 19 years	0.2137	0.07–0.72	Tang *et al.*, 2022 ((66))
19 years and older	0.365	0.273–0.418	Tang *et al.*, 2022; Assumption ((66))
**Neurologic sequelae utility decrement (per year)**			
Younger than 19 years	0.2456	0.16–0.49	Tang *et al.*, 2022 ((66))
19 years and older	0.5278	0.22–0.783	Tang *et al.*, 2022; Assumption ((66))

Abbreviations: AOM, acute otitis media; IPD, invasive pneumococcal disease; pCAP, pneumococcal community acquired pneumonia; Pneu-C, pneumococcal conjugate vaccine

To compare the three vaccination strategies, we conducted a sequential cost-effectiveness analysis ((38)). In short, the three vaccination strategies were ordered from lowest to highest cost. Incremental costs and QALYs gained were compared between a given strategy and the next less costly strategy. A vaccination strategy was considered dominated if at least one other vaccination strategy was expected to result in additional QALYs gained at a lower cost.

### Scenario and sensitivity analyses

We conducted a scenario analysis to estimate the potential impact of vaccine-derived indirect effects on ICERs by including an exponential decline in PD incidence caused by Pneu-C-15 specific serotypes (i.e., 22F and 33F) and Pneu-C-20 specific serotypes (i.e., 8, 10A, 11A, 12F, 15B, 22F and 33F) across all age groups. We included an exponential decline ranging from 0%–50%, with effects beginning one year after the vaccination program was implemented and taking five to 10 years to reach maximum effect.

We also evaluated the cost-effectiveness of the three vaccination strategies in a higher cost, higher PD incidence setting such as that observed in the circumpolar region ((18,67)). Age-specific relative risks were calculated by comparing IPD incidence in Yukon, Northwest Territories and Nunavut to all of Canada (including the territories) ((18)). A relative measure of the increased cost associated with medical care in Yukon, Northwest Territories and Nunavut compared to all of Canada was extracted from an economic analysis of pneumococcal vaccines in older adults ((53)). We applied these multipliers to *S. pneumoniae*-attributed health outcomes and relevant costs in our base case analysis.

In addition to a probabilistic sensitivity analysis, we conducted deterministic sensitivity analyses to examine the robustness of the base case findings to our assumptions. First, we examined the impact of varying key model parameters in our base case in a one-way sensitivity analysis. Parameters were varied across a range of values (Tables 1-4). Second, given the uncertainty of the prices of Pneu-C-15 and Pneu-C-20, we conducted a two-way sensitivity analysis. We varied the incremental price of Pneu-C-15 and Pneu-C-20 to be up to 50% higher than the assumed price of Pneu-C-13. Third, we lowered the incidence of pCAP and AOM in our model, reflective of data from British Columbia ((19)); data from Ontario informed our base case analysis. Fourth, we lowered the number of AOM cases projected to be prevented by replacing Pneu-C-13 with Pneu-C-15 or Pneu-C-20. This reflects the lower AOM incidence attributed Pneu-C-15 and Pneu-C-20 vaccine serotypes in the United States ((68)).

Although Canada does not have a set cost-effectiveness threshold, we used two common thresholds, $30,000 per QALY and $60,000 per QALY, in our scenario and sensitivity analyses for illustrative purposes ((69,70)).

Our study follows the Professional Society for Health Economics and Outcomes Research (ISPOR) Consolidated Health Economic Evaluation Reporting Standards (CHEERS) 2022.

## Results

**Figure 2:**
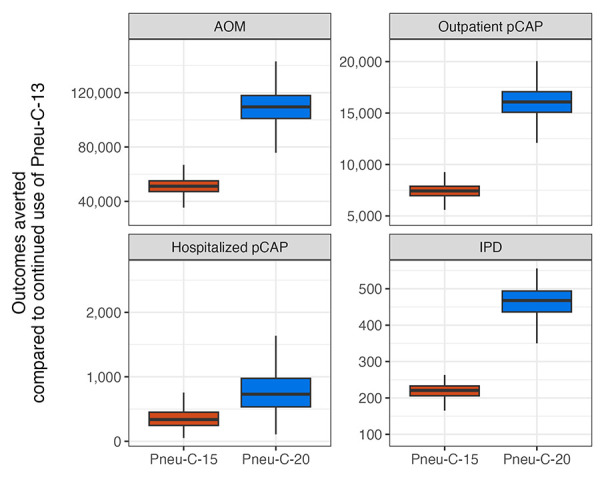
Outcomes averted in all age groups compared to continued use of Pneu-C-13 over 10 years in the base case scenario^a^ Abbreviations: AOM, acute otitis media; IPD, invasive pneumococcal disease; pCAP, pneumococcal community acquired pneumonia; Pneu-C, pneumococcal conjugate vaccine ^a^ Results are shown for 10,000 model simulations

The use of Pneu-C-15 and Pneu-C-20 averted additional *S. pneumoniae*-attributable health outcomes over 10 years compared to the continued use of Pneu-C-13 (**Figure 2**). On average, Pneu-C-15 averted an additional 221 (interquartile range [IQR]: 206–233) IPD cases, 337 (IQR: 533–976) hospitalized pCAP cases, 7,428 (IQR: 6,965–7,885) outpatient pCAP cases and 51,143 (IQR: 47,184–55,089) AOM cases in the Canadian population compared to the continued use of Pneu-C-13 in our base case. The Pneu-C-20 vaccine averted an additional 468 (IQR: 436–494) IPD cases, 730 (IQR: 533–976) hospitalized pCAP cases, 16,084 (IQR: 15,082–17,071) outpatient pCAP cases and 109,527 (IQR: 101,054–117,926) AOM cases compared Pneu-C-13.

**Figure 3:**
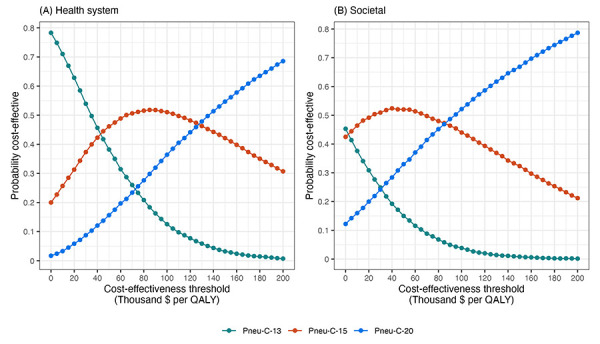
Percent of simulations for which each vaccination strategy was the optimal strategy for a given cost-effectiveness threshold in the base case from the health system and societal perspectives, in the absence of indirect effects Abbreviations: Pneu-C, pneumococcal conjugate vaccine; QALY, quality-adjusted life year

From the health system perspective, replacing Pneu-C-13 with Pneu-C-15 is expected to save an average of 497 QALYs and cost an additional $30 million over 10 years (**Table 5**). Replacing Pneu-C-13 with Pneu-C-20 is expected to save an average of 1,039 QALYs and cost an additional $103 million over ten years. From the societal perspective, Pneu-C-15 is expected to cost an additional $9 million and Pneu-C-20 is expected to cost an additional $60 million over 10 years compared to the continued use of Pneu-C-13. From the health system perspective, Pneu-C-15 is most likely to be the optimal strategy at cost-effectiveness threshold ranges of $43,000 to $127,000 per QALY (**Figure 3**). Above $127,000 per QALY, Pneu-C-20 is most likely to be the optimal strategy. From the societal perspective, Pneu-C-15 is most likely to be the optimal strategy at cost-effectiveness threshold ranges of $3,000 to $86,000 per QALY, and Pneu-C-20 is most likely to be the optimal strategy at thresholds above $86,000 per QALY.

**Table 5:** Mean quality-adjusted life years lost, cost and incremental cost-effectiveness ratios for the base case scenario, in the absence of indirect effects

Strategy	Effect (QALYs lost)	Cost ($, millions)	Sequential ICER ($/QALY)
**Health system perspective**			
Pneu-C-13	229,769	4,945	-
Pneu-C-15	229,272	4,975	58,823
Pneu-C-20	228,730	5,048	135,289
**Societal perspective**			
Pneu-C-13	229,769	432,243	-
Pneu-C-15	229,272	432,252	18,272
Pneu-C-20	228,730	432,303	93,416

Abbreviations: ICER, incremental cost-effectiveness ratio; Pneu-C, pneumococcal conjugate vaccine; QALY, quality-adjusted life year; -, not applicable

**Figure 4:**
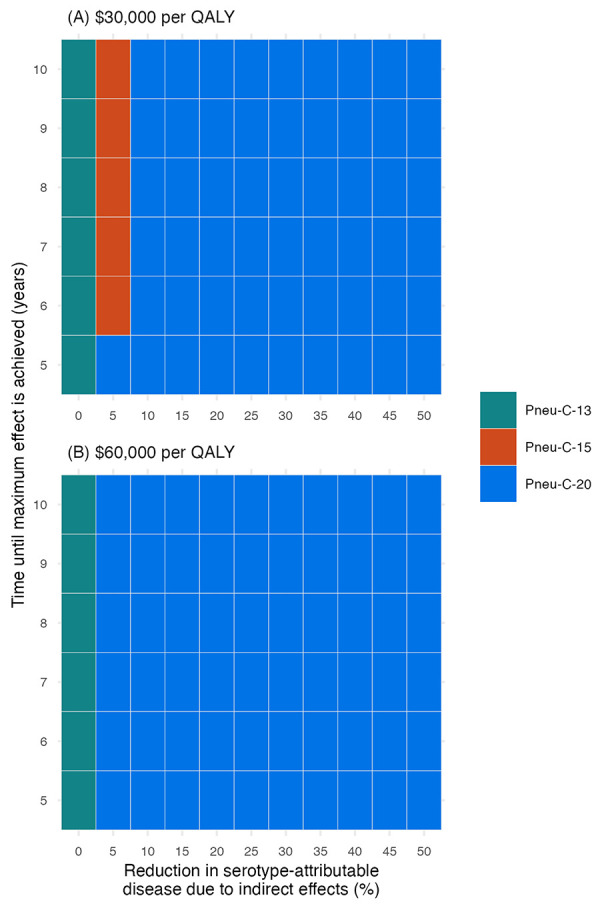
Impact of a reduction in serotype-attributable disease due to indirect vaccine effects on the optimal vaccination strategy at $30,000 and $60,000 per QALY from a health system perspective^a^ Abbreviations: Pneu-C, pneumococcal conjugate vaccine; QALY, quality-adjusted life year ^a^ Results are a function of the percent reduction in serotype-attributable disease due to indirect effects and the time until the maximum effect is achieved

The inclusion of indirect effects leads to lower ICERs because of the resulting reduction in PD among population members who did not receive the vaccine (**Figure 4**). At a cost-effectiveness threshold of $30,000 per QALY, a 5% reduction in PD caused by the additional serotypes contained in Pneu-C-15 over a six-year period would result in Pneu-C-15 being the optimal strategy. At a cost-effectiveness threshold of $30,000 per QALY, a 10% or greater percent decrease in PD over a five-year period caused by the additional serotypes contained in Pneu-C-20 results in Pneu-C-20 being the preferred strategy. From the societal perspective, even smaller indirect effects would result in Pneu-C-15 or Pneu-C-20 being the optimal strategy.

**Figure A1:**
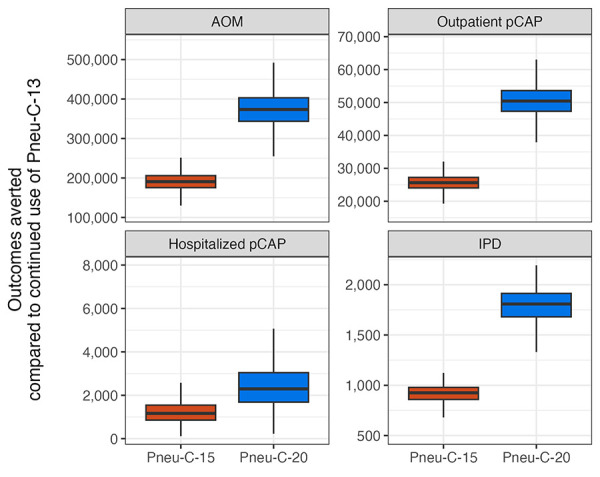
Outcomes averted in all age groups compared to continued use of Pneu-C-13 over 10 years in the higher cost and higher pneumococcal disease incidence scenario^a^ Abbreviations: AOM, acute otitis media; IPD, invasive pneumococcal disease; pCAP, pneumococcal community acquired pneumonia; Pneu-C, pneumococcal conjugate vaccine ^a^ Results are shown for 10,000 model simulations

In a higher cost and higher PD incidence setting, on average, Pneu-C-15 averted an additional 925 (IQR: 859–979) IPD cases, 1,116 (IQR: 855–1,545) hospitalized pCAP cases, 25,638 (IQR: 24,055–27,254) outpatient pCAP cases and 190,760 (IQR: 175,466–205,884) AOM cases on average over 10 years compared to the continued use of Pneu-C-13 (**Appendix, ****Figure A1**). The Pneu-C-20 vaccine averted an additional 1,808 (IQR: 1,680–1,914) IPD cases, 2,294 (IQR: 1,683–3,039) hospitalized pCAP cases, 50,446 (IQR: 47,333–53,624) outpatient pCAP cases and 373,543 (IQR: 343,610–403,099) AOM cases compared Pneu-C-13. The Pneu-C-20 vaccine dominates (i.e., is less costly and more effective than) Pneu-C-13 and Pneu-C-15 from the both the health system and societal perspectives (Appendix, **Table A1**). The Pneu-C-20 vaccine is dominant, with lower costs and fewer QALYs lost than the current strategy (i.e., Pneu-C-13) and Pneu-C-15.

**Figure 5:**
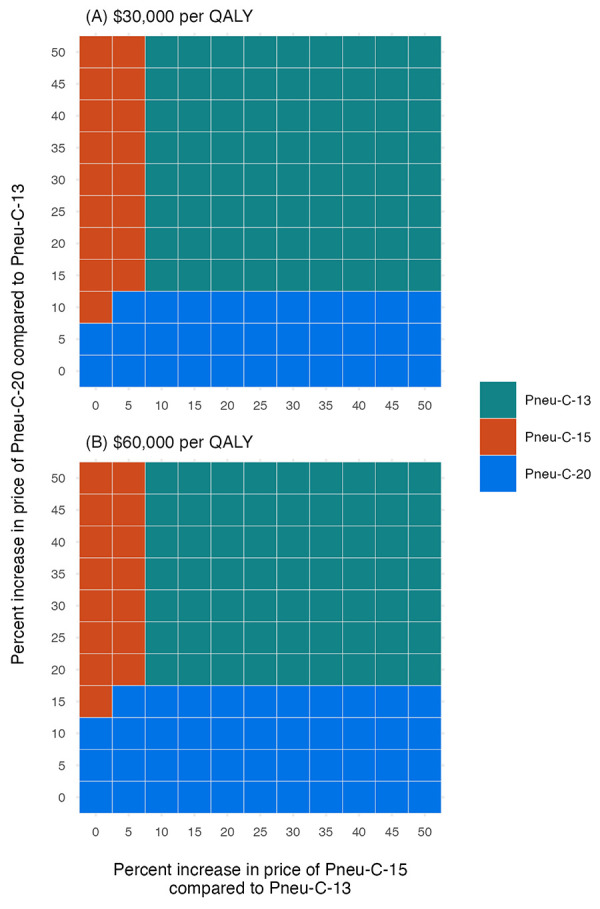
Sensitivity analysis of vaccine costs^a,b^ Abbreviations: Pneu-C, pneumococcal conjugate vaccine; QALY, quality-adjusted life year ^a^ Incremental cost-effectiveness ratios were calculated for a range of prices per dose for Pneu-C-15 and Pneu-C-20, ranging from 0%–50% higher than the price of Pneu-C-13 ^b^ The optimal strategy was identified for cost-effectiveness thresholds of $30,000 and $60,000 per QALY from the health system perspective

Our base case conclusions relied on several assumptions that we examined in sensitivity analyses. In our one-way sensitivity analysis of model parameters, vaccine price was the most influential parameter (not shown). When the relative vaccine prices of Pneu-C-15 and Pneu-C-20 compared to Pneu-C-13 were increased compared to their base case values, Pneu-C-13 remained the strategy with the lowest ICER (**Figure 5**). At a $30,000 per QALY threshold, Pneu-C-15 was the optimal strategy when the relative price increase of Pneu-C-15 was 5% or less than the price of Pneu-C-13. The Pneu-C-20 vaccine was the optimal strategy when the relative price increase of Pneu-C-20 was 10% or less than the price of Pneu-C-13. At a $60,000 per QALY threshold, Pneu-C-15 or Pneu-C-20 was the optimal strategy if the relative price increases for Pneu-C-15 or Pneu-C-20 were 5% or 15% or less than the price of Pneu-C-13, respectively. A lower incidence of pCAP and AOM led to sequential ICERs of over $100,000 per QALY for Pneu-C-15 and over $200,000 per QALY for Pneu-C-20. Additionally, an AOM serotype distribution more similar to the United States, which differs from the serotype distribution of IPD in Canada, results in sequential ICERs of over $100,000 per QALY for Pneu-C-15 and Pneu-C-20.

## Discussion

We conducted an economic evaluation to estimate the health impact and cost-effectiveness of replacing Pneu-C-13 with Pneu-C-15 or with Pneu-C-20 for routine use in the paediatric population in Canada. Our base case results found that both Pneu-C-15 and Pneu-C-20 prevented additional cases of IPD, pCAP and AOM compared to the continued use of Pneu-C-13. In our base case, Pneu-C-15 would require a threshold of $58,823 per QALY from the health system perspective and $18,272 per QALY from the societal perspective to be considered cost effective. The Pneu-C-20 vaccine would require a threshold of $135,289 per QALY from the health system perspective and $93,416 per QALY from the societal perspective to be considered cost effective. In contrast, with the inclusion of moderate indirect vaccine effects (e.g., a reduction of 5% or greater in serotype-attributable PD), both Pneu-C-15 and Pneu-C-20 could be considered cost effective at thresholds under $30,000 per QALY from the health system and societal perspectives. In a higher cost and higher PD incidence setting, Pneu-C-20 dominates the other vaccination strategies.

A recent comparative analysis of three cost-utility models conducted in the United States compared Pneu-C-20 to either Pneu-C-15 or Pneu-C-13 using a 3+1 schedule in children younger than two years of age ((71)). It showed similar trends as our analysis, with Pneu-C-20 expected to result in the largest gain in health outcomes compared to the other vaccines. From the societal perspective, results varied across the three included models, with ICERs for Pneu-C-20 ranging from dominant to $162,700 per QALY compared to Pneu-C-15. The models included in this analysis were all static but differed in structure, analytic time horizon, assumptions about indirect protection effects and key parameters, further highlighting the sensitivity of these model-based economic evaluation results to model assumptions and input parameters ((13)).

The estimated cost-effectiveness of the different conjugate vaccines was driven, in part, by the presence or absence of indirect effects. After the introduction of Pneu-C-13 in paediatric populations, IPD incidence caused by the serotypes in the vaccine decreased in all age groups ((7,8)), but overall IPD incidence in the population did not substantially decrease ((10)). In several countries including Canada, the introduction of pneumococcal conjugate vaccines (i.e., Pneu-C-7, Pneu-C-10 and Pneu-C-13) resulted in increases in the incidence of IPD caused by serotypes not included in the vaccines across all ages ((72,73)). In our base case analysis, we conservatively did not include indirect effects, given the uncertainty of herd immunity effects and serotype replacement. In our scenario analysis, we modelled indirect effects as a decline in pneumococcal disease in the broader population not receiving the higher valency conjugate vaccines.

Uncertainty about vaccine price in the Canadian context adds complexity to the interpretation of our results, given how influential the prices of Pneu-C-15 and Pneu-C-20 were on the estimated ICERs. In sensitivity analysis, we showed that at lower incremental prices compared to the price per dose of Pneu-C-13, both higher valency vaccines can be cost-effective options. Our analysis provides an indication of the prices at which either vaccine may become the optimal strategy based on commonly used thresholds.

## Limitations

Because we used a static model, our approach did not fully capture the transmission dynamics associated with herd immunity effects and serotype replacement. Future economic evaluations of pneumococcal conjugate vaccination should consider using dynamic models to inform cost-utility analyses to better capture these effects ((74)).

Additionally, our economic evaluation focused on children beginning their pneumococcal vaccination series. We did not assess the cost-effectiveness of the three strategies among children who were mid-way through their vaccine series, and we did not assess the impact of a potential catch-up program. Our estimates of the incidence of PD included children at both low and high risk of PD. We did not identify the optimal vaccination strategy independently among children at higher risk for PD outside of a higher cost setting.

## Conclusion

Our study provides evidence of the impact Pneu-C-15 and Pneu-C-20 could have on reducing the burden of PD in Canada compared to the continued use of Pneu-C-13. Although ICERs were relatively high in the base case analysis, at lower vaccine prices and/or in the presence of indirect effects in the broader population following vaccine introduction, both vaccines have the potential to improve health in a cost-effective manner.

## Appendix

**Table A1:** Mean quality-adjusted life years lost, cost, and incremental cost-effectiveness ratios for the higher cost and higher pneumococcal disease incidence scenario, in the absence of indirect effects

Strategy	Effect (QALYs lost)	Cost ($, millions)	Sequential ICER ($/QALY)
**Health system perspective**			
Pneu-C-20	15,794	541,539	-
Pneu-C-15	15,819	543,513	Dominated by Pneu-C-20
Pneu-C-13	15,897	545,613	Dominated by Pneu-C-20
**Societal perspective**			
Pneu-C-20	541,539	445,465	-
Pneu-C-15	543,513	455,579	Dominated by Pneu-C-20
Pneu-C-13	545,613	445,760	Dominated by Pneu-C-20

Abbreviations: ICER, incremental cost-effectiveness ratio; Pneu-C, pneumococcal conjugate vaccine; QALY, quality-adjusted life year; -, not applicable
